# Dr. Sutherland on Undiluted Creasote in Burns

**Published:** 1842-05-07

**Authors:** John Sutherland


*Undiluted Creasote in Burns.* By John Sutherland, M.D.—It has been my intention for some time to send you a short communication on the use of undiluted creasote in burns, but I have delayed doing so in the expectation that I might be able to try the remedy in a greater number and variety of cases, so as to obtain an estimate of its real value. In the 22d number of the Lancet, however, there is a communication from Dr. Mitchell, of Dublin, on the subject, in which the author requests information from other members of the profession who may have used it, and in consequence of this I shall state shortly the result of my experience.

About two years ago I accidentally dropped some burning sealing-wax on my hand; I removed it as quickly as possible, and applied to the surface a drop of creasote from a bottle which happened to be beside me; it afforded immediate relief, and in less than five minutes the pain was gone. No sensible vesication took place, but in a few days a cuticle peeled off, and left a dry, reddish surface beneath. A short time after this one of my servants burned the palm of her hand by laying hold of a hot iron; in a few minutes creasote removed the pain, and I heard nothing more of it. I have used it in ten or a dozen similar cases, and I have always found it remove the pain in a very short time. The effects I had observed induced me to bring the subject before the Medical Institution of Liverpool (as I find by the minutes) on the 1st of April, 1841, in the hope that such members as had opportunity might give the remedy a trial. I have had no experience of its use in severe injuries, and on this point I can bear no testimony, but in the common domestic burn or scald it has appeared to me so useful that I have recommended its being kept in families for the purpose of immediate application.—*Ibid.*

